# Notes and News

**Published:** 1894-05-26

**Authors:** 


### Notes and News.

Collected and Communicated.

MEETINGS OF THE WEEK.

Royal Hospital for Incurables, Putney, Festival Dinner, Hotel Metropole, Thursday, May 31st.

Middlesex Hospital, Festival Dinner, Hotel Metropole, Friday, June 1st.

St. Mary's Hospital, Festival Dinner, Hotel Metropole, Friday, June 1st.

North London Consumption Hospital, Festival Dinner, Hotel Metropole, Saturday, June 2nd.

Final arrangements have been made that the Prince and Princess of Wales will lay the foundation-stone of the Royal Alexandra Hospital at Rhyl, after their visit to Carnarvon to attend the national Eisteddfod meetings.

The Princess of Wales has consented to open the new Home for Incurables at Streatham on the afternoon of Monday, July 2nd, on which occasion Her Royal Highness will be accompanied by the Prince of Wales, and it is hoped by other members of the Royal Family. An urgent appeal for £5,000 is being made, so that the board may report to Her Royal Highness on that occasion that the home has been opened free of debt.

We are glad to see that our contemporary, the *British Medical Journal*, is not inclined to leave the matter relating to the well-being of the helpless little inhabitants of the Forest Gate Schools alone. It does not appear that any effective control has been arranged which secures that the children's food shall be adequate in quantity and in quality. If the scandal convinces the Local Government Board that large establishments are very undesirable for the boarding and education of poor children, the sufferings of the children at Forest Gate will not have been wholly evil. Only those who have had experience within these large rearing establishments can fully understand how easy it is to mismanage and at the same time throw dust in the eyes of partially experienced committees and the public.

The Duke of Saxe-Coburg and Gotha paid a visit to the Charing Cross Hospital last week, and recorded in the visitors' book his satisfaction with everything he had seen. He also attended a performance of the Orchestral Society of the hospital.

Two Northern hospitals at any rate cannot complain that their claims have been forgotten in regard to legacies and donations. The Wakefield Hospital will become possessed of between £20,000 and £25,000 through the will of Mr. S. Fozard Harrison, a solicitor of Wakefield; and the Huddersfield Infirmary is to receive a generous donation of £1,000 from Sir John Ramsden, Bart., towards the enlargement of its buildings. From a Christian mission in the town £11 has also been received by the institution.

The Medical Defence Union held a meeting on the 8th inst., to consider the action of the council in reference to the litigation between Drs. Collie and Bloxham. Mr. Bloxham was the first applicant for assistance to the Union, but he withdrew his application, and that of Dr. Collie, made subsequently, was accepted. Mr. Bloxham issued a writ against the Union for maintenance, which is still standing; also before the meeting he served a writ for libel and slander against the president, Professor Victor Horsley. Whatever other effect may result arising out of the case, it is likely that the council will be more chary in espousing the cause of members of the profession than before.

The new convalescent home for the Foresters of the Midland Counties has just been opened. The scheme is a very interesting one, as it is entirely the work of members of the Order of Foresters, who pay a penny a week each towards the support of a convalescent home. There are over 27,000 subscribers. Previously the order supported a smaller home for 15 inmates. The building just opened will contain double that number, and the site admits of an extension allowing for 60 beds. In addition to the weekly subscription, the Foresters each gave a shilling towards the new building. Of the £3,250 expended on the building and furnishing, £1,100 yet remains to be paid off. The home is beautifully situated at Clent, near Stourbridge, and the comfort of the patients has been well thought out.

We have received an appeal for that excellent charity the Children's Fresh-Air Mission. We are reminded that 10s. will secure two weeks' residence in the country for a child from the densely populated districts of Holborn, Clerkenwell, and St. Luke's. Last year the society were able to send 2,630 children into the country. It would be distressing, indeed, if fewer could enjoy the benefits of such an opportunity this season. There is an organiser in each centre to which the children are sent, to whose supervision much of the success of the work is due. Gifts of clothing, as well as money, are gratefully accepted for the children. Donations and subscriptions should be sent to the Hon. Treasurer, Mr. Walter Hazell, St. Peter's Schools, Onslow Street, Clerkenwell Road, E.C.

